# Validation of the National Health And Nutritional Survey (NHANES) single-item self-reported sleep duration against wrist-worn accelerometer

**DOI:** 10.1007/s11325-021-02542-6

**Published:** 2021-11-30

**Authors:** Paul H. Lee

**Affiliations:** Department of Health Sciences, George Davies Centre, University of Leicester, University Road, Leicester, LE1 7RH UK

**Keywords:** Actigraphy, Measurement, Questionnaire, Sleep

## Abstract

**Purpose and methods:**

This study aimed to validate the single-item sleep duration question used in the National Health And Nutritional Survey (NHANES), “How much sleep do you usually get at night on weekdays or workdays (hours)?”, against a wrist-worn accelerometer (ActiGraph GT3X +) in waves 2011–2012 and 2013–2014 among an adult population aged 20 or above (*n* = 8,438, mean age 49.7, 48% male).

**Results:**

The accelerometer-measured and self-reported sleep duration were 6.01 (SD 1.48) and 6.88 (SD 1.40) h/day, respectively, representing a 0.87 h/day of over-reporting (SD 1.90, *p* < 0.001). Such an over-reporting was observed in all subgroups, where the over-reporting ranged from 0.72 (those aged 41–50) to 1.13 h/day (those aged 71 or above). The correlation between accelerometer-measured and self-reported sleep duration was low (*ρ* = 0.14, *p* < 0.001).

**Conclusions:**

The associations between sleep duration and other health outcomes identified using NHANES data should be further tested using more accurate and valid measures of sleep duration.

**Supplementary Information:**

The online version contains supplementary material available at 10.1007/s11325-021-02542-6.

## Introduction

Measuring habitual sleep duration is essential in observational studies as it is correlated with many other health outcomes [1–3]. Three methods can be used to measure habitual sleep, including sleep questionnaire (single point self-reporting of habitual sleep duration), sleep diary that reports time in bed and wake up time for a representative period of time (usually 7 consecutive days), and accelerometry using an electronic device that measures the movement pattern of the subject under investigation, with the wake-sleep pattern determined by prolonged non-movement. All these methods are validated against the gold standard of sleep measurement in a laboratory condition, polysomnography [4–6], although discrepancies have been observed between these sleep measures and polysomnography, and the validity varied among subjects with sleep disorders [7]. Among these three measurements, accelerometry is the only objective measurement, and it has become more popular in sleep research due to its decreasing cost and ability to collect additional data such as physical activity and both electric and outdoor sunlight exposure [8]; therefore, accelerometry is also being regarded as a standard of sleep measurement in a free-living condition [9, 10], albeit with an overestimation in sleep duration and underestimation in wake after sleep onset and sleep onset latency [11].

Given its low cost and ease of administration, self-reported sleep duration remains a popular choice in observational studies. The sleep diary has a better validity than a sleep questionnaire as a diary is completed on a day-by-day basis and can capture the sleep variation of the respondents. The sleep diary was shown to have less than a 15-min difference in measuring sleep duration compared with accelerometry, albeit moderately correlated (*r* = 0.4–0.6) [12, 13]. However, the sleep diary introduced a burden to its respondents, and systematic bias existed due to the unavoidable difference between actual sleep onset time/wake up time and time at completing the diary. Therefore, self-reported questionnaires are still being widely used albeit with questionable validity. For instance, the National Health And Nutritional Survey (NHANES), which surveyed a US-representative sample of around 5,000 individuals each year, used a single-item question “How much sleep do you usually get at night on weekdays or workdays (hours)?” from wave 2005–2006 to wave 2017–2018 to measure sleep duration. Data collected using this question had been widely used as a measure of habitual sleep duration, and a number of studies correlated this with other health outcomes in NHANES [1, 2]. The single-item question “On average, how many hours of sleep do you get in a 24-h period?” used in Behavioral Risk Factor Surveillance System (BRFSS) has been validated in a subsample of 300 participants [14], but the validity of the NHANES question has not been validated. As a quality assurance procedure, this study aimed to validate the single-item total sleep duration question used in NHANES against a wrist-worn accelerometer (ActiGraph GT3X +) in waves 2011–2012 and 2013–2014 among an adult population aged 20 or above. The results may help evaluate the methodological quality of these existing studies using NHANES data.

## Participants and methods

### Participants

The complete details of the NHANES recruitment procedure can be found on the NHANES official website, https://wwwn.cdc.gov/nchs/nhanes/continuousnhanes/overview.aspx?BeginYear=2011. A total of 11,329 participants aged 20 + were recruited in NHANES 2011–2012 and 2013–2014, and only those who provided valid data on self-reported and accelerometer-measured sleep duration (defined in the “Measurement” section) were included in the present analysis.

### Measurement

#### Self-reported sleep duration

Participants were asked “How much sleep do you usually get at night on weekdays or workdays (hours)?”. Participants responded with an integer value between 2 and 11, and responses of 12 h or more were coded to 12. I regarded sleep duration of 2 h/day as outliers and were removed from the analysis (*n* = 31).

#### Accelerometer-measured sleep duration

The complete details of the accelerometer procedure can be found on the NHANES official website (https://wwwn.cdc.gov/nchs/data/nhanes/2011-2012/manuals/2012-Physicial-Activity-Monitor-Procedures-Manual-508.pdf). In short, participants were invited to wear an accelerometer (ActiGraph GT3X + , https://actigraphcorp.com/) on their non-dominant wrist at the day of the examination, continue to wear it 24 h a day for 7 consecutive days, and remove it on the morning of the 9th measurement day. The accelerometer measured the acceleration with an 80 Hz frequency, and the epoch length was set at 1 min. Each of the measured minute was classified as either wake, sleep, non-wear, or unknown according to the signal power, variance of the orientation, and change of the orientation using a machine learning algorithm [15]. For the current analysis, sleep onset was defined as a consecutive sleeping period of at least 15 min, and a sleep period ended if a consecutive waking period of at least 15 min were recorded. The non-wear and unknown status of accelerometer data were not used in the current analysis. Sleep duration was calculated as the difference between the sleep onset and sleep offset. A sensitivity analysis was conducted to test the robustness of this parameter by computing the total sleep duration using 5-min, 10-min, and 20-min criteria. To align with the self-reported sleep duration, accelerometer data at weekends (i.e., Friday–Saturday and Saturday–Sunday nights) were removed from the analysis.

### Data analysis

All accelerometer-measured sleep duration of < 3 or > 12 h/day were regarded as outliers and removed from the analysis. Paired sample *t*-test and Pearson correlation were used to examine the difference and correlation between self-reported sleep duration and accelerometer-measured sleep duration, respectively. Mean and SD of accelerometer-measured sleep duration across all levels of self-reported sleep duration were reported. The self-reported sleep duration was classified as underestimation, accurate estimation, and overestimation if the difference between the corresponding accelerometer-measured sleep duration was smaller than − 0.5 h/day, between − 0.5 and 0.5 h/day, and larger than 0.5 h/day, respectively. Bland–Altman plot was used to evaluate the agreement of self-reported sleep duration and accelerometer-measured sleep duration. All statistical analysis was conducted using R 4.0. The R syntax for accelerometer data processing is available as supplementary material.

## Results

A total of 8,438 participants (mean age 49.7, SD 17.6) were included in the present analysis. On average, 2.8 (SD 1.2) valid accelerometer-measured sleeping episodes (i.e., sleep duration between 3 and 12 h) were provided by the participants, and the intra-class correlation coefficient was 49.5%. Table 1 shows the demographic characteristics and sleep duration of the participants. The sample was uniform across age and gender, and most of them were Non-Hispanic Whites (40.8%) and Blacks (23.4%). More than half of them had at least some college or AA degree (56.0%) and were married (50.8%). A large over-reporting was observed in the average daily sleep duration. The accelerometer-measured and self-reported sleep duration were 6.01 (SD 1.48) and 6.88 (SD 1.40) h/day, respectively, representing a 0.87 h/day of over-reporting (SD 1.90, *p* < 0.001). Such an over-reporting was observed in all subgroups, where the over-reporting ranged from 0.72 h (those aged 41–50) to 1.13 h/day (those aged 71 or above). The correlation between accelerometer-measured and self-reported sleep duration represented a small but positively, statistically significant effect size (*ρ* = 0.14, *p* < 0.001). A similar pattern was observed among all subgroups, with correlations ranging from 0.02 (separated) to 0.19 (those who graduated from college or above).

**Table 1:** Accelerometer-measured sleep duration (h/day) of the participants (*n* = 8,438)

Variable	Accelerometer-measured sleeping time, 5-min definition^1^ (mean (SD))	Difference with self-reported sleeping time# (SD)	Correlation	Accelerometer-measured sleeping time, 15-min definition^2^ (mean (SD))	Difference with self-reported sleeping time# (SD)	Correlation	Accelerometer-measured sleeping time, 20-min definition^3^ (Mean (SD))	Difference with self-reported sleeping time# (SD)	Correlation
Total	5.07 (1.11)	1.82 (1.72)	0.07***	5.68 (1.41)	1.21 (1.88)	0.12***	5.49 (1.59)	1.44 (2.00)	0.11***
Age									
Below 30	5.28 (1.18)	1.70 (1.76)	0.02	5.75 (1.42)	1.23 (1.87)	0.08**	5.51 (1.57)	1.46 (1.97)	0.08*
31–40	5.24 (1.09)	1.56 (1.68)	0.05	5.72 (1.39)	1.08 (1.87)	0.08**	5.60 (1.63)	1.27 (2.07)	0.02
41–50	5.11 (1.09)	1.56 (1.64)	0.12***	5.68 (1.40)	1.00 (1.84)	0.12***	5.44 (1.56)	1.32 (1.88)	0.12***
51–60	5.00 (1.10)	1.73 (1.68)	0.10***	5.58 (1.37)	1.16 (1.86)	0.10***	5.40 (1.73)	1.37 (1.99)	0.12***
61–70	4.92 (1.06)	1.97 (1.63)	0.14***	5.62 (1.44)	1.28 (1.85)	0.17***	5.42 (1.55)	1.54 (1.88)	0.20***
71 or above	4.81 (1.07)	2.49 (1.74)	0.07*	5.73 (1.46)	1.55 (1.94)	0.14***	5.60 (1.69)	1.72 (2.17)	0.11**
Gender									
Male	4.99 (1.10)	1.88 (1.70)	0.06***	5.63 (1.41)	1.24 (1.86)	0.12***	5.44 (1.57)	1.48 (1.97)	0.12***
Female	5.14 (1.12)	1.76 (1.73)	0.08***	5.73 (1.41)	1.18 (1.89)	0.11***	5.54 (1.61)	1.40 (2.02)	0.10***
Race									
Mexican American	5.06 (1.07)	1.90 (1.68)	0.06	5.64 (1.40)	1.32 (1.84)	0.12***	5.49 (1.60)	1.46 (1.95)	0.13***
Other Hispanic	5.05 (1.08)	1.77 (1.66)	0.06	5.70 (1.37)	1.13 (1.81)	0.09*	5.46 (1.52)	1.40 (1.92)	0.09
Non-Hispanic White	5.20 (1.15)	1.85 (1.76)	0.05	5.83 (1.43)	1.21 (1.89)	0.11***	5.62 (1.60)	1.50 (2.02)	0.09***
Non-Hispanic Black	4.85 (1.05)	1.75 (1.75)	0.07**	5.43 (1.43)	1.18 (1.97)	0.10***	5.31 (1.65)	1.32 (2.07)	0.13***
Non-Hispanic Asian	5.05 (1.10)	1.88 (1.57)	0.04	5.67 (1.34)	1.26 (1.74)	0.07*	5.42 (1.48)	1.54 (1.91)	0.001
Others	5.20 (1.17)	1.47 (1.71)	0.18**	5.65 (1.30)	1.04 (1.83)	0.15*	5.56 (1.48)	1.12 (1.84)	0.23**
Education									
Less than 9th grade	4.87 (1.00)	2.18 (1.73)	0.06	5.54 (1.39)	1.49 (1.89)	0.12**	5.29 (1.52)	1.76 (2.04)	0.13*
9th–11th grade	4.99 (1.16)	1.88 (1.86)	0.08**	5.69 (1.50)	1.19 (2.05)	0.09**	5.56 (1.66)	1.35 (2.14)	0.102*
High school graduate/GED	5.03 (1.09)	1.87 (1.77)	0.06**	5.63 (1.41)	1.28 (1.91)	0.13***	5.46 (1.57)	1.49 (1.96)	0.17***
Some college or AA degree	5.10 (1.13)	1.68 (1.71)	0.10***	5.68 (1.46)	1.91 (1.96)	0.08***	5.50 (1.64)	1.35 (2.06)	0.08**
College graduate or above	5.18 (1.11)	1.78 (1.57)	0.06**	5.78 (1.32)	1.18 (1.62)	0.17***	5.53 (1.52)	1.45 (1.82)	0.09**
Marital status									
Married	5.08 (1.10)	1.85 (1.62)	0.08***	5.70 (1.38)	1.23 (1.77)	0.06	5.50 (1.58)	1.50 (1.90)	0.10**
Widowed	4.89 (1.03)	2.12 (1.72)	0.13***	5.72 (1.51)	1.29 (2.00)	0.10*	5.68 (1.66)	1.21 (2.11)	0.12***
Divorced	5.07 (1.18)	1.67 (1.81)	0.11***	5.61 (1.46)	1.14 (1.97)	0.11*	5.39 (1.61)	1.48 (2.08)	0.11*
Separated	4.94 (0.99)	1.62 (1.75)	0.09	5.57 (1.46)	0.99 (2.07)	0.05	5.44 (1.54)	0.99 (2.04)	0.02
Never married	5.12 (1.14)	1.77 (1.79)	0.06**	5.68 (1.43)	1.22 (1.93)	0.11***	5.54 (1.63)	1.35 (2.05)	0.12***
Living with partner	5.14 (1.13)	1.70 (1.94)	0.15	5.64 (1.40)	1.18 (2.09)	0.12***	5.27 (1.46)	1.65 (2.16)	0.02

^#^All differences significant at 0.1% level

^1^Sleep onset is defined as a consecutive sleeping period of at least 5 min, and a sleep period will end if a consecutive waking period of at least 5 min were recorded

^2^Sleep onset is defined as a consecutive sleeping period of at least 15 min, and a sleep period will end if a consecutive waking period of at least 15 min were recorded

^3^Sleep onset is defined as a consecutive sleeping period of at least 20 min, and a sleep period will end if a consecutive waking period of at least 20 min were recorded

**Fig. 1:**
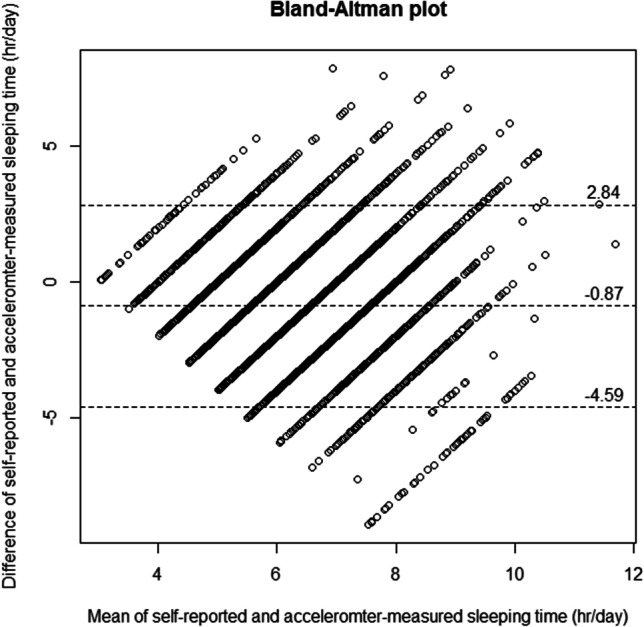
Bland-Altman plot for the agreement of self-reported sleep duration and accelerometer-measured sleep duration. The mean bias and 95% limits of agreement were 0.87 and (-4.59, 2.84), respectively

Table 2 shows the distribution of the self-reported sleep duration, as well as the accelerometer-measured sleep duration across all levels of self-reported sleep duration (3–12 h/day). While there was a positive association between the sleep duration measured by accelerometer and self-report, the association was weak, and the mean accelerometer-measured sleep duration (h/day) across the groups differed by less than 2 h. For self-reported sleep duration of 5 h or less, the self-reported sleep duration overestimated the objectively measured sleep duration, while the self-report underestimated those who have an objectively measured sleep duration of 6 h or more. Figure 1 shows the Bland–Altman plot for these two measurements, where their large discrepancy was revealed by the wide range of the 95% limits of agreement (− 4.59, 2.84).

**Table 2:** Accelerometer-measured sleep duration (h/day) of the participants across different levels of self-reported sleep duration (h/day) (*n* = 8,438)

Self-reported sleep duration (h/day)	Frequency	Percentage	Underestimation^1^ (*n* = 1810, 21.5%)	Accurate estimation^2^ (*n* = 1630, 19.3%)	Overestimation^3^ (*n* = 4998, 59.2%)	Mean of accelerometer-measured sleep duration (h/day)	SD of accelerometer-measured sleep duration (h/day)
3	61	0.7	53 (86.9%)	8 (13.1%)	0 (0.0%)	5.39	1.49
4	350	4.1	227 (79.1%)	61 (17.4%)	12 (3.4%)	5.69	1.44
5	854	10.1	431 (50.5%)	243 (28.5%)	180 (21.1%)	5.74	1.51
6	1961	23.2	573 (29.2%)	561 (28.6%)	827 (42.2%)	5.86	1.39
7	2254	26.7	308 (13.7%)	484 (21.5%)	1462 (64.9%)	6.03	1.42
8	2285	27.1	152 (6.7%)	237 (10.4%)	1896 (83.0%)	6.18	1.52
9	431	5.1	12 (2.8%)	31 (7.2%)	388 (90.0%)	6.38	1.54
10	180	2.1	3 (1.7%)	5 (2.8%)	172 (95.6%)	6.41	1.61
11	15	0.2	1 (6.7%)	0 (0.0%)	14 (93.3%)	7.20	1.92
12	47	0.6	0 (0.0%)	0 (0.0%)	47 (100.0%)	5.99	1.58

^1^Self-reported sleep duration—accelerometer-measured sleep duration <  − 0.5 h/day

^2^ − 0.5 h/day ≤ self-reported sleep duration—accelerometer-measured sleep duration < 0.5 h/day

^3^Self-reported sleep duration—accelerometer-measured sleep duration ≥ 0.5 h/day

Table 1 shows the results of the sensitivity analysis. The accelerometer-measured sleep duration using the 5-min, 10-min, and 20-min criteria were 5.07 (SD 1.39), 5.68 (SD 1.41), and 5.49 (SD 1.59), respectively. They were mildly correlated (*ρ* = 0.37–0.79), and they all have small but significant correlation with self-reported sleep duration (*ρ* = 0.07–0.12). Similar patterns were found using different definitions of accelerometer-measured sleep onset and awake (5-min, 15-min, and 20-min definitions). The results of this sensitivity analysis supported that the conclusions drawn using the main study were robust to the criterion used to define sleep onset and awake.

## Discussion

This study shows that self-reported single-item total sleep duration was only weakly associated with the sleep duration measured by a wrist-worn accelerometer (ActiGraph GT3X +), and participants over-reported their sleep duration by approximately 52 min per day with a wide 95% limits of agreement (− 2 h 50 min, 4 h 35 min). Therefore, the validity of this single-item sleep duration measurement and the validity of studies examining the associations between sleep duration and other health outcomes using NHANES data are questionable. Results obtained from research assessing sleep duration using this single-item question should be further tested using more accurate and valid measures of sleep duration. For analysis of sleep using NHANES data, the accelerometer data should be used instead given its validity and ability to measure other sleep parameters including sleep efficiency and wake after sleep onset.

Results of this study are not without limitations. The reference measure of sleep duration in the current study, the actigraphy, has its own limitations. Accelerometers are found to overestimate sleep duration by about 5–15 min in adult populations [5, 10, 11], indicating that the over-reporting of sleep duration might be more than the 52 min found in the current study. Note that the machine learning algorithm used to detect sleep duration in the current study has not been validated in a free-living condition among a general population. However, visual inspection of the accelerometer data from several participants by the authors confirmed the validity of this algorithm. There were no data on the time lag between self-reported and objectively measured sleep duration; thus, its effect on the validity of self-reported sleep duration could not be evaluated. Furthermore, as no data were available on the participants’ working pattern, a Monday to Friday pattern was assumed, and the accelerometer-measured sleep duration extracted may not have represented participants who worked on weekends.

The single-item question on sleep duration was used in NHANES from waves 2005–2006 to waves 2015–2016. However, only waves 2011–2012 and 2013–2014 of NHANES were used here as these were the only two waves where respondents concurrently wear an accelerometer so that correlating of the two measures are feasible. The same sleep duration question was implemented in waves 2015–2016. In NHANES 2017–2018, another question was added to collect self-reported sleep duration during weekends. In fact, for sleep duration, two types of questionnaire are used in the literature; the first type is a single-item question that asks the respondents the average sleep duration per night (e.g., NHANES and BRFSS [14]), and the second type is a two-item questionnaire that asks the sleep onset time and wake up time, and the sleep duration is determined by their difference (e.g., Pittsburgh Sleep Quality Index, PSQI [16]). In NHANES 2019–2020, instead of asking the total sleep duration, the second type of questionnaire was used. However, no concurrent objective measurement of sleep duration was available for 2015–2016 onwards, and the validity of the new sleep questions could not be evaluated.

In BRFSS [14], a US community sample comparable to NHANES, both self-reported and accelerometer-measured sleep duration, was 7 h/day. In the current sample, the self-reported sleep duration was 7 h/day and accelerometer-measured 6 h/day. Assuming that BRFSS and NHANES had a similar target population, it is possible that the accelerometer algorithm was biased and underestimated the actual sleep duration. However, no other measurements of sleep duration were available for the NHANES sample, and this postulation could not be examined. Also, the BRFSS subsample analyzed in the aforementioned study may not be comparable to NHANES as it was a small (*n* = 300) and geographically limited (Upstate New York region) study.

With the NHANES that surveyed a US-representative sample of *n* ~ 5,000 each year, much population-level research could be conducted, for example, sleep patterns in sub-groups and longitudinal change in sleep patterns. A simple analysis was performed here on the average sleep duration across different age groups, gender, ethnic groups, and education level (Table 1). The current research serves as a starting point of the above possibilities by providing the validity of the single-item sleep duration question.

## Supplementary Information

Below is the link to the electronic supplementary material.

Supplementary file1 Supplementary material 1. R syntax for NHANES accelerometer data processing (TXT 6 KB)

## Data Availability

The dataset(s) supporting the conclusions of this article is(are) available in the NHANES website (https://wwwn.cdc.gov/nchs/nhanes/).

## Code availability

The R syntax is available as a supplementary material.
